# RNAs on the Go: Extracellular Transfer in Insects with Promising Prospects for Pest Management

**DOI:** 10.3390/plants10030484

**Published:** 2021-03-04

**Authors:** Dulce Santos, Simon Remans, Stijn Van den Brande, Jozef Vanden Broeck

**Affiliations:** Research Group of Molecular Developmental Physiology and Signal Transduction, Division of Animal Physiology and Neurobiology, Department of Biology, KU Leuven, Naamsestraat 59, 3000 Leuven, Belgium; simon.remans@kuleuven.be (S.R.); stijn.vandenbrande@kuleuven.be (S.V.d.B.); jozef.vandenbroeck@kuleuven.be (J.V.B.)

**Keywords:** crop protection, extracellular vesicle, exosome, intercellular communication, interkingdom, interspecies, non-coding RNA, pest control, RNA binding protein, RNA delivery, RNA interference (RNAi), small RNA

## Abstract

RNA-mediated pathways form an important regulatory layer of myriad biological processes. In the last decade, the potential of RNA molecules to contribute to the control of agricultural pests has not been disregarded, specifically via the RNA interference (RNAi) mechanism. In fact, several proofs-of-concept have been made in this scope. Furthermore, a novel research field regarding extracellular RNAs and RNA-based intercellular/interorganismal communication is booming. In this article, we review key discoveries concerning extracellular RNAs in insects, insect RNA-based cell-to-cell communication, and plant–insect transfer of RNA. In addition, we overview the molecular mechanisms implicated in this form of communication and discuss future biotechnological prospects, namely from the insect pest-control perspective.

## 1. Introduction: Regulatory RNAs and Insect-Plant Interactions

RNAs exist in a wide variety of structures and sizes, well suited to regulate a multitude of processes. Regulatory RNAs, also referred to as non-coding RNAs, do not contribute directly to protein synthesis but function at various control levels to modulate gene expression. These molecules act both at the transcriptional and post-transcriptional levels, by mediating chromatin modulation, regulating alternative splicing, inducing suppression of translation, or directing the degradation of target transcripts [1].

Eukaryotic regulatory RNAs are broadly classified into long (≥200 nt) and small (≤200 nt). While numerous of the so-called long non-coding RNAs are described to regulate gene expression at various levels, it has recently been shown that some might, in fact, have coding functions [1,2]. Nonetheless, long non-coding RNAs and the mechanisms by which they exert their functions are still poorly characterized and deserve further research efforts. On the other hand, small RNA (sRNA)-based regulatory mechanisms are well established. In particular, the discovery of the RNA interference (RNAi) mechanism in animals resulted in a Nobel Prize and motivated a boom of comprehensive studies unveiling the functional role of these molecules in post-transcriptional silencing [3,4,5,6]. In short, during RNAi, sRNAs of approximately 18–30 nt are incorporated into an RNA-induced silencing complex (RISC), which is then directed to a target transcript via Watson–Crick base pairing. Subsequently, an Argonaute (Ago) protein within RISC acts to inhibit or degrade the target transcript, resulting in suppressed gene expression [7,8].

Classification of sRNAs relies on their biogenesis mechanisms, size, complementarity to the target, associated proteins, and main regulatory processes in which they are involved. Based on these, several sRNAs are recognized among eukaryotes, of which two are common to plants and animals: microRNAs (miRNAs) and small interfering RNAs (siRNAs). In broad terms, miRNAs originate from the processing of endogenous stem-loop RNA precursors and act to regulate the expression of endogenous genes. In turn, siRNAs originate from long double-stranded RNA (dsRNA) structures and mainly function in the protection against viruses and transposons [9,10,11]. While many other sRNA types are distinguished, within and beyond the formerly described classes, these are not discussed in the context of the current review.

Although the mechanisms by which they act are not as extensively investigated as in eukaryotes, regulatory RNAs are also present in Archaea and Bacteria. In this regard, the RNA chaperone Hfq is well described to play a central role in several RNA-based regulatory systems in prokaryotes [12,13,14,15,16,17]. Furthermore, prokaryotic Ago proteins have been shown to contribute to some forms of RNA-guided gene regulation [18,19,20]. In addition, the CRISPR-Cas (clustered regularly inter-spaced short palindromic repeats and associated genes) system has attracted a lot of attention due to its exceptional potential for RNA-guided genome editing. In fact, this mechanism led to the 2020 Nobel Prize in Chemistry [21,22]. While prokaryotic regulatory RNAs differ from their eukaryotic counterparts in several aspects, such as size range and associated pathways, their contribution to regulatory mechanisms in multiple processes is also evident [23,24,25].

While regulatory RNAs and their pathways are mostly investigated at the intracellular level, discoveries regarding a role of extracellular RNAs in cell-to-cell communication are now making the scene. Numerous are the examples of interspecies and even interkingdom RNA-based communication, especially those concerning host–parasite/symbiont interactions. The study of RNA-based transfer of information promises not only to bring fundamental insights into a novel layer of regulatory complexity, but also to open doors to a handful of biotechnological applications [26,27,28].

Insects are the largest and most diverse group of animals on Earth. They form an important component of the ecosystems and affect many aspects of human life. A major part of insects’ impact occurs via their interaction with plants; for instance, some species are important pollinators, while others are agricultural pests and vectors of plant diseases [8,29,30]. In this scope, insects and their host plants have gone through a long coevolution and thereby developed several known forms of communication via specific effector proteins, volatiles, and other chemicals [31,32]. Recently, growing evidence suggests the existence of RNA-based cell-to-cell communication in insects and in plant–insect interactions. Understanding the mechanisms of intercellular RNA transfer in insects, as well as of the communication between Animalia and Plantae, promises to contribute to the development of novel technologies. These are crucial to cope with insect-related challenges, and valuable examples are RNAi-directed pest control strategies. In this manuscript, we review the current state of the art concerning RNA-based communication in and between insects, as well as the RNA transfer between plants and insects. In addition, we discuss the possible underlying transfer mechanisms and the related biotechnological prospects.

## 2. Extracellular RNA-Based Communication

The presence of RNA molecules in extracellular environments, mainly of sRNAs, has been systematically reported. Throughout the past decade, it became evident that RNA molecules mediate an important layer of communication, even between diverse and phylogenetically distant organisms [28,33,34]. In plants, the role of mobile RNAs in intercellular communication has been well-established [35,36]. In animals, extracellular RNAs are extensively described in mammalian biofluids. These molecules are known to be secreted followed by functional uptake into recipient cells [37,38,39,40,41,42,43,44,45,46,47,48,49,50,51,52,53,54,55,56]. In invertebrates, the presence of extracellular RNAs has been demonstrated in arthropods and nematodes, with a limited number of studies demonstrating functional RNA transfer [57,58,59,60,61,62,63,64,65,66,67,68,69,70,71,72,73,74,75,76,77,78,79,80,81,82]. Below, we review the current knowledge on extracellular RNAs in insects, establishing the distinct levels of this communication: intercellular/intraindividual, interindividual, interspecies, and interkingdom (insect–plant).

### 2.1. Intercellular/Intraindividual

In insects, little more than a handful of studies report extracellular RNAs and their (potential) role in intercellular/intraindividual communication, namely in beetles and flies: (i) extracellular sRNA populations have been observed in the hemolymph of the korean rhinoceros beetle, *Allomyrina dichotoma* [67]; (ii) miRNAs were detected in cell-free hemolymph of *Drosophila melanogaster* flies, and some were demonstrated to differentially accumulate with age [62]; (iii) several sRNA populations have been found in the extracellular medium of the *D. melanogaster* cell lines S2R+ and D17-c3 [63]; (iv) extracellular miRNAs have been identified in medium of cultured *D. melanogaster* cells, namely of S2 and Cl8 cell lines, with indications for differential secretion [65]; (v) in *Drosophila* flies, extracellular siRNAs have been shown to contribute to systemic antiviral immunity [64]; (vi) extracellular siRNAs were demonstrated to spread the RNAi signal in red flour beetle, *Tribolium castaneum* cultured cells [66]; and (vii) in the Colorado potato beetle, *Leptinotarsa decemlineata*, dsRNA has been identified in the extracellular medium of cultured cells previously treated with dsRNA [68]. These findings are summarized in Table 1.

### 2.2. Interindividual

Besides serving cell-to-cell communication within single organisms, RNA molecules have the potential to mediate interactions between individuals. This is particularly studied in insects, where interindividual RNA-based communication has been shown in social species. The first finding regards the honey bee, *Apis mellifera*, where caste determination has been shown to be influenced by miRNAs in nurse bee secretions [70]. More recently, a transmissible RNA pathway across generations, via the hemolymph and followed by secretion in the jellies, has been demonstrated [71,72]. In addition, miRNAs have been identified in the trophallactic fluid of the Florida carpenter ant, *Camponotus floridanus,* contributing to the concept of interindividual RNA transfer in social insects [73]. Table 1 resumes these reports.

### 2.3. Interspecies

Functional RNA transfer between different species is also currently described in animals. This is broadly termed interspecies RNA-based communication, and the so-far described examples mainly refer to host–parasite interactions [74,75,77]. A fascinating example is reported in insects and concerns the parasitic wasp *Cotesia vestalis* and its host *Plutella xylostella*, the diamondback moth. Specifically, the developing wasp larva has the capacity to release teratocyte cells into the host body. These cells then secrete extracellular vesicles (EVs) containing the miRNA Cve-miR-281. On another front, the wasp larva also releases the associated *C. vestalis* bracovirus, which expresses the Cve-miR-novel22 miRNA in the host. Remarkably, both miRNAs influence the host’s ecdysone cascade to delay its development, hereby granting the parasite additional time to develop [74]. In addition, sRNAs have been identified in saliva of mosquitos and other blood-feeding arthropods, allowing speculation about functions of these molecules in modulating host response [59,60,61,82]. In line with this, parasitic nematodes were shown to secrete sRNA molecules, which, in some cases, have been proven to regulate host biology [77,78,79,80]. The reports on (potential) interspecies RNA-based communication in insects are briefed in Table 1. Noteworthy, interspecies RNA communication is also reported within Plantae. The studied examples mainly regard parasitic plants from the genus *Cuscuta* and their hosts, where bidirectional RNA transfer has been demonstrated [83,84,85,86,87,88,89,90]. Furthermore, *Cuscuta* miRNAs have been reported to target *Arabidopsis* genes [91]. In addition, unidirectional movement of small RNAs from shoots to roots in a soybean–common bean (*Glycine max-Phaseolus vulgaris*) grafting system has been recently demonstrated [92].

### 2.4. Interkingdom

There are known cases of RNA transfer crossing the three domains of life and several examples of interkingdom RNA-based communication between plants or insects and their microorganisms. Within Eukarya, functional RNA transfer has been reported between plants (Plantae) or insects (Animalia) and their fungal (Fungi) or protist (Protista) pathogens [28,93,94,95,96,97]. In this scope, the currently available knowledge points towards the existence of functional RNA transfer systems between insects and plants, as addressed in the following sections.

#### 2.4.1. Insect to Plant RNA-Based Communication

Until now, very little is known regarding RNA transfer from insects to plants, with only three reported studies. The first indication for a possible insect–plant functional RNA transfer was obtained in the green peach aphid, *Myzus persicae*. In this study, reduced aphid fecundity was observed when the insect fed on *Arabidopsis* mutants for the miRNA pathway. This led to speculation that processing of aphid miRNAs by the plant might mediate aphid infestation [98]. Later, sRNAs from the phloem-feeding whitefly *Bemisia tabaci* have been identified both in tissue and phloem of the tomato plant [99]. Interestingly, more recently, *M. persicae* aphids were demonstrated to transfer the *Ya* transcript to their host plants, where it moves to distal leaves. This transcript was identified as a long non-coding RNA virulence factor, with functional studies demonstrating that it promotes aphid fecundity [100].

#### 2.4.2. Plant to Insect RNA-Based Communication

The presence and functionality of dietary RNAs in animals have been a matter of debate over the last decade [101,102,103,104,105,106]. Nevertheless, in insects, increasing functional evidence points towards the natural existence of some insect–plant RNA-based communication levels. Several studies identify plant-derived RNAs in herbivorous insects, and some even report functionality. The presence of *Cucumis melo* miRNAs has been shown in the cotton–melon aphid, *Aphis gossypii*, demonstrating that plant miRNAs are stable in the insect body [107]. In the silkworm, *Bombyx mori*, mulberry miRNAs were identified in both hemolymph and tissues [108]. Later, sorghum miRNAs were identified in the greenbug, *Schizaphis graminum,* and yellow sugarcane miRNAs in the yellow sugarcane aphid, *Sipha flava*. In silico target prediction indicated the involvement of these miRNAs in processes important for aphid fecundity, suggesting a role in decreasing aphid infestation [109]. Another study identified miRNAs of *Brassica oleracea* in the gut of *M. persicae,* the green peach aphid. The predicted miRNA targets in the insect were mainly carbohydrate transport and metabolism, RNA processing and modification, and nuclear structure genes [110]. Plant-derived miRNAs were also shown to be present in the hemolymph of *P. xylostella*, the diamondback moth. Importantly, two of the most abundant identified plant miRNAs were validated to target *P. xylostella* hemocyanin domain containing genes, that are known to play important roles in the hemolymph of arthropods. Moreover, other highly abundant plant miRNA was demonstrated to influence the pupal development and egg-hatching rate [111,112]. Remarkably, plant miRNAs found in honeybee larval food contribute to caste determination. Specifically, plant miRNAs are enriched in beebread in comparison with royal jelly, some of which were demonstrated to prevent larval differentiation into queens and to induce development into worker bees. Feeding miR162a to larvae resulted in worker bee phenotypes, with similar results in *D. melanogaster* larvae [113]. This miRNA targets *A. mellifera* TOR, a key player in cast development [113,114]. Table 2 summarizes the studies reviewed in this section. 

#### 2.4.3. Engineered Plant-to-Insect RNA Transfer

Adding to the previously described observations, it is relevant that plant–insect functional transfer of RNA can be engineered to occur. In short, host plants can be genetically modified to express specific RNA molecules targeting key insect genes. When the insect feeds on the plant, these RNA molecules enter the insect body, are taken up by recipient cells, and induce gene silencing by RNAi. In the context of insect pest control, there are several examples of transgenic plants designed to induce RNAi in insects, a process commonly designated as host-induced gene silencing. The first successful studies on achieving resistance to a pest–insect via transgenic plants expressing dsRNA date to 2007 [115,116]. Since then, multiple proofs of this concept have been reported [117,118,119,120,121,122,123,124,125,126,127,128,129,130,131]. Often, long dsRNA molecules are expressed. However, in planta expression of insect miRNAs has been suggested to be a good option as well [132,133,134,135]. In addition, a plant-expressed insect miRNA precursor was shown to be more effective than expressing the miRNA itself [136]. The authors suggested this was due to the fact that insect miRNA precursors are not processed by the plant, since they lack Drosha, an important nuclear processing enzyme of insect miRNA biogenesis [136]. In addition, expression of RNAi-inducing molecules in the chloroplast instead of in the cellular cytoplasm, via modification of the chloroplast genome, revealed to be quite promising [126,137,138,139]. Since no RNAi-processing machinery is present in this organelle, higher accumulation of the RNA molecules in the plant is obtained, resulting in a more effective RNAi response in the insect [126,137,138,139]. Nevertheless, this approach is not always effective, suggesting that, in some cases, RNA molecules from the chloroplast are not transferred to the insects [140]. Since recently, the first RNAi-based pest-resistant plant has been available on the market. It is named SmartStax Pro^®^ and was developed by Monsanto and Dow Agrosciences [141]. This illustrates the economical relevance of this field and emphasizes the need for understanding the natural mechanisms of plant–insect RNA transfer, possibly contributing to the development of efficient pesticides based on exogenous RNA.

## 3. Mechanisms of RNA Transfer

Transfer of information via extracellular RNAs has been seen to occur in insects. In addition, bidirectional plant–insect RNA transfer has been reported and, in some cases, the RNA signal was shown to be functional. This was demonstrated both in the context of specific (possibly coevolved) plant–insect interactions, as well as of genetically engineered plants, to exert RNA-based insect pest control (see Section 2). Nevertheless, little is known regarding the mechanisms by which mobile RNAs spread in insects, or between plant and insect cells. To date, three main routes for cell-to-cell RNA based communication have been proposed, namely via transfer of naked RNA, RNA bound to RNA-binding proteins (RBPs), and RNA-containing extracellular vesicles (EVs).

### 3.1. Naked RNA

In plants, naked RNAs, i.e., not protected by RNA binding proteins or encapsulated in EVs, can be transferred over short and long distances [36,142,143,144]. Short range transfer of naked sRNAs in plants occurs via the plasmodesmata, by the space between the plasma membrane and the desmotubule, and by the desmotubule itself, which connects the endoplasmic reticulum of the two adjacent cells [144,145,146,147]. Similarly, in fungi, naked sRNA can move from cell-to-cell within short distances through the septal pore [144]. In mammals, transfer between adjacent cells can be mediated by gap junctions. In this respect, siRNA- and miRNA-mediated transfer via gap junctions has been described [148,149,150,151,152,153,154,155,156,157]. To our knowledge, the transfer of RNA molecules via gap junctions has not been investigated in other groups of animals. However, in the nematode *Caenorhabditis elegans*, transmembrane systemic RNA interference defective (SID) proteins act as channels for intercellular RNA movement [158,159,160,161]. In insects, short range transport of dsRNA and RISC components through nanotube-like structures was suggested between *Drosophila* cells [162]. However, whether these structures might effectively transfer (naked) RNAs between adjacent insect cells remains to be demonstrated. Taken together, these studies suggest that similar structures might function for short range RNA transfer in plants, fungi and animals.

In both plants and fungi, apoplastic movement of naked sRNA can also occur. In this case, naked sRNAs are secreted from the plasma membrane and move throughout the cell wall to extracellular spaces, where they can then enter both neighboring or distant cells [144,145,146,147].

Plants also transfer naked sRNAs via the phloem, using the vascular system to spread these molecules throughout the plant to distant cells [144,146,147]. In addition, it is noteworthy that several reports indicate the transfer of naked sRNA between plants and fungi [96,163,164,165], indicating bidirectional interkingdom RNAi between plants and fungi. Specialized infection structures of fungi and parasitic plants, termed haustoria, may act as a gateway for sRNA transfer between host and pathogen at the plant–plant and plant–fungi levels [91].

In human plasma, naked extracellular RNAs are rapidly degraded [166]. Similarly, naked RNA molecules are rapidly degraded in insect biofluids [8,167,168,169,170,171]. Nevertheless, it is by now clear that stable RNA molecules circulate in animal extracellular fluids (see Section 2). Together, these facts contribute to the idea that mobile RNAs in animal biofluids require protection form degradation in order to be functionally transferred.

### 3.2. RNA Associated with RNA Binding Proteins (RBPs)

In plants, RBPs are established to mediate short- and long-range RNA transport. The *Cucurbita maxima* Phloem Small RNA-Binding Protein 1 can bind sRNAs, transferring them between cells, both via the plasmodesmata and the phloem [172,173]. Furthermore, other RBPs have been identified in the phloem of different plants [174,175,176]. Interestingly, Ago proteins have also been suggested to be implicated in sRNA transfer in plants [177,178]. In addition, recently, a conserved family of sRNA-binding proteins–Small RNA-Binding Protein 1 family—that function in intercellular transfer of sRNAs has been identified in the phloem of several plants [179].

**Figure 1:**
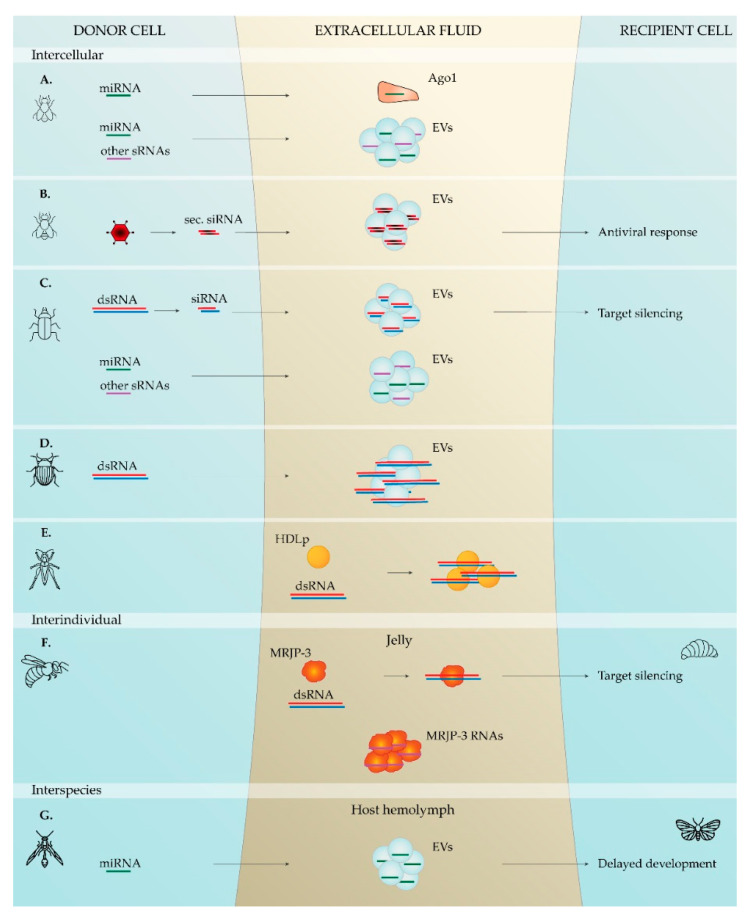
Summary of the known mechanisms involved in the presence of extracellular RNAs and their functional transfer in insects. (**A**) *D. melanogaster*—miRNAs were identified in immunoprecipitates of extracellular Ago proteins and in EVs from the culture medium of *D. melanogaster* cells, namely the Cl8 and the S2 cell lines [65]. In addition, miRNAs and other sRNA populations were identified in EVs from the culture medium of the *D. melanogaster* S2R+ and D17-c3 cell lines [63]. (**B**) *D. melanogaster*—EVs from *D. melanogaster* hemocytes contain secondary viral siRNAs, synthesized from viral DNA. These EVs circulate in the hemolymph and functionally spread these viral siRNAs, thereby inducing systemic antiviral immunity [64]. (**C**), *T. castaneum*—dsRNA-derived siRNAs are found in EVs from the culture medium of *T. castaneum* TcA cells. These siRNA-containing EVs trigger RNAi in recipient cells. miRNAs and other sRNAs were also identified in these EVs [66]. (**D**) *L. decemlineata*—dsRNA was identified in EVs from the culture medium of *L. decemlineata* Lepd-SL1 cells, previously treated with dsRNA [68]. (**E**) *S. gregaria*—upon microinjection in the hemocoel, dsRNA binds to lipophorins in the hemolymph [169,192]. (**F**) *A. mellifera*—Major Royal Jelly Protein 3 (MRJP-3) binds to dsRNA in the jelly, protecting it from degradation and enhancing its uptake. MRJP-3 also binds single-stranded RNA and several populations of sRNAs in the jellies [71,72]. In parallel, ingested dsRNA was shown to spread in the hemolymph and to be secreted in worker and royal jellies, via which it passes to larvae, triggering target silencing [71]. (**G**) *C. vestalis/P. xylostella*—Larva of the parasitic wasp *C. vestalis* secretes teratocyte cells into its host, *P. xylostella*. These teratocytes secrete miRNA-containing EVs that enter host’ cells, where the miRNAs induce a delay in host development [74].

In 2008, Mitchell and colleagues demonstrated that extracellular sRNAs present in human plasma are protected from degradation due to their association with certain entities [166]. In line with this, most mammalian plasma miRNAs are associated with Ago proteins [180,181,182]. Interestingly, Neuropilin-1 has been reported to be a receptor for miRNA–Ago complexes [183]. Nevertheless, due to the remarkable extracellular stability reported for some Ago proteins, it is often suggested that extracellular RNA–Ago complexes are by-products of cell death [180,181,184]. In the nematode *Heligmosomoides bakeri*, secondary siRNAs are loaded into an extracellular Ago protein, and this complex is subsequently secreted in EVs, suggesting a role of this Ago protein in mediating the selective sorting of sRNAs in EVs in this species [79]. In the fruit fly, extracellular miRNAs have been shown to be stably present in the hemolymph, and an in vitro study with *Drosophila*-derived cell lines verified the presence of extracellular miRNAs associated with an Ago protein [62,65], suggesting that Ago proteins might also confer sRNA stability in insects (Figure 1).

Besides Ago proteins, the association of sRNAs to lipoproteins has been demonstrated as well. Lipoproteins have been shown to be associated with miRNAs, and high-density lipoproteins (HDLs) can functionally transfer miRNAs to recipient cells [185]. Furthermore, miRNA-delivery mediated by HDL was shown to be dependent on scavenger receptor class B type I [185]. Since then, other reports have emphasized the role of HDLs in intercellular RNA transfer, as well as the potential use of these lipoproteins as therapeutic-delivery vehicles [185,186,187,188,189,190,191]. Interestingly, insect and other arthropod lipoproteins were demonstrated to adhere to dsRNA, suggesting a potential role of these proteins in mediating RNA-based communication in this phylum [169,192,193] (Figure 1).

Additionally to lipoproteins and Ago proteins, other animal proteins have been suggested to bind RNA in the extracellular environments. Particularly, mammalian Nucleophosmin1 was demonstrated to bind miRNAs and protect them from nuclease degradation [194]. In insects, more specifically in honeybees, a secreted RBP named Major Royal Jelly Protein 3 (MRJP-3) binds to RNA in jelly, protecting it from degradation and enhancing its uptake [72] (Figure 1).

### 3.3. RNA-Containing Extracellular Vecicles (EVs)

EVs form a heterogeneous group consisting of exosomes, microvesicles and apoptotic bodies. Although long viewed as part of cellular waste disposal pathways, it is by now clear that EVs can functionally transfer their content (RNA, DNA, lipid, and protein) to recipient cells [195].

Despite previous debate regarding plant cell wall preventing formation and function of EVs, recent evidence shows that EVs are also produced by these organisms [97,165,196,197,198]. In addition, plant EVs have been shown to contain RNA [197,199,200,201], and selective sRNA loading in EVs has been observed [202]. Moreover, the transfer of sRNAs within EVs from plantae to fungi has been recently demonstrated [97]. Interestingly, specific RBPs, including Ago proteins, have been suggested to facilitate the packaging of RNAs into EVs in plants [178,203].

In 2007, a first study demonstrating that EVs mediate intercellular communication in mammalian cell lines, by transferring functional RNA from donor to recipient cells, was reported [37,38]. Since then, a myriad of reports indicate EV-mediated intercellular communication in mammals [39,40,41,42,43,44,45,46,47,48,49,50,51,52,53,54,55,56,204,205,206,207,208,209]. Currently, increasing evidence points towards the ubiquitous presence of RNA-containing EVs in animals, as suggested by studies in the nematodes *C. elegans* [57,58,69,76], *Heligmosomoides polygyrus*, *Litomosoides sigmodontis* [77], *Brugia malayi* [78], *H. bakeri,* and *Trichuris muris* [80]; in the ticks *Ixodes Ricinus* and *Haemaphysalis longicornis* [59,82]; as well as in the red swamp crayfish, *Procambarus clarkia* [81]. Also in insects, several reports from recent years suggest the involvement of EVs in a common mechanism for functional RNA transfer between cells. RNA-containing EVs have been reported in the fruit fly, namely in the hemolymph [62,64] and in cultured cells [63,65]; as well as in beetles, specifically in the hemolymph of *A. dichotoma* [67] and in cell lines of *T. castaneum* [66] and *L. decemlineata* [68]. Moreover, EV-specific miRNA profiles have been shown in *Drosophila* [62,65]. Noteworthy, functional transfer of RNA within EVs was demonstrated in three studies. First, hemocyte-derived EVs containing secondary viral siRNAs confer systemic RNAi antiviral immunity in *D. melanogaster* [64]. Second, in *T. castaneum* cell culture, siRNA-containing EVs spread an RNAi-based silencing signal to recipient cells [66]. Third, EVs of the parasitic wasp *C. vestalis* deliver miRNAs to its host *P. xylostella*, which target its ecdysone cascade, resulting in a developmental delay [74]. Likewise, the parasitoid wasp *Leptopilina boulardi* injects vesicles in its host *Drosophila*. Although transfer of RNA has not been investigated in this case, these EVs—venosomes—were shown to interfere with the hosts’ defense [210]. Moreover, EVs present in bee pollen, honey, and royal jelly were shown to function in the antibacterial properties of these products [211]. Together, these studies suggest that EVs function in transport of molecules in and between insects, including their RNA cargo (Figure 1).

Two potential RNA selective sorting mechanisms have been proposed in mammals. First, miRNAs packaged into EVs were demonstrated to contain specific nucleotide motifs with which specific ribonucleoproteins interact to allow selective sorting [212,213,214,215,216,217,218,219]. Moreover, several membrane proteins involved in EV biogenesis are also involved in selective miRNA sorting [50,220,221,222]. Second, certain miRNA post-transcriptional modifications appear to contribute to their selective sorting, as 3′ end uridylated miRNAs were found overrepresented in EVs while 3′ end adenylated miRNAs were relatively enriched intracellularly [223].

EVs are produced by all domains of life and are considered part of an ancient mechanism for RNA export [224,225]. In fact, several reports describe EV-mediated RNA transfer, within and among animals, plants, fungi and microbes [11,28,33,34,144,197,198,225,226,227]. Although further detailed research is needed to investigate potential mechanisms of RNA transfer between insects and plants, the current knowledge indicates EVs as promising candidates. Figure 1 summarizes the findings regarding RNA transfer mechanisms in insects.

## 4. Biotechnological Prospects

To sustain the currently growing food demand, agricultural pests are one of the many challenges that are faced. Pest insects destroy 18–20% of the annual global crop production, which is estimated at $470 billion [228]. The classical approach of combatting agricultural insect pests is via non-selective conventional insecticides. However, their limited target species selectivity has serious disadvantages, including a detrimental impact on environment and human health [229,230,231,232]. In fact, these concerns are increasing and have led to a ban in some commonly used insecticides [233,234,235]. In addition, some pest insect populations are already resistant against many of the conventionally used insecticides [236,237,238,239,240,241].

RNA-mediated insect gene silencing is a promising tool to contribute to highly specific pest control strategies. Nevertheless, the effect of RNA is not equally efficient in every insect, with high levels of variability between different species and even between different populations of the same species. RNA degradation by gut nucleases and extreme pH conditions upon ingestion is appointed as a reason for inefficient RNA-based silencing in several insect species. In addition, inefficient or absent functional RNA uptake systems in the gut, as well as the lack of an efficient systemic RNAi spreading mechanism from the gut to the rest of the insect body, are also considered potentially important limiting factors [8,168,242,243]. RNA might be available for ingestion by the insect via different approaches, namely via expression by genetically modified plants (see Section 2.4.3) or by exogenous in planta application. As such, several RNA insect delivery systems have been proposed, meant to exert RNA protection and mediate intracellular delivery. Well-known examples are based on nanoparticles, liposomes, RBPs, bacteria and viruses, among others [8]. In this scope, natural systems of RNA transfer within insects, as well as from plants to insects, represent key fundamental concepts that might lead the development of more efficient RNA-based insect gene silencing strategies.

In mammals, both RBPs and EVs have been explored for efficient cellular delivery of nucleic acids. These approaches are mainly explored in humans, in the context of targeted drug delivery therapeutics. Interesting examples are mammalian lipoproteins, which are often proposed for use in human siRNAs delivery [244,245,246,247,248,249]. In addition, a high percentage of mammalian extracellular miRNAs are bound to Ago proteins, and pre-assembled siRNA-Ago complexes, delivered via different carriers, can increase the gene silencing effect in mice [180,181,182,250]. Interestingly, insect lipoproteins (i.e., lipophorins) are known to bind exogenous dsRNA in the hemolymph [169,192], and Ago proteins have been identified in the extracellular medium of cultured insect cells [65]. Hence, these proteins may be promising candidates for design of exogenous RNA insect delivery systems, highlighting the importance of investigating natural RNA transfer mechanisms.

Besides RBPs, many studies appoint EVs as promising human drug-delivery vehicles [206,251,252]. In fact, engineering of EVs to deliver nucleic acid based therapeutics is already being explored in the market [253,254]. Considering the role of EVs in RNA-based intercellular, interspecies, and interkingdom communication, such structures might hold great potential for RNA-based pest management [226]. In addition, since increasing evidence indicates the role of EVs in transferring RNA molecules in insects (see Section 3.3), it is exciting to consider the development of EV-based RNA-delivery systems to control pests via exogenous RNA. Although RBP- and EV-based crop protection systems are still to be explored, these may lead to promising strategies for the future.

To ensure environment-friendly and biosafe insecticides, specificity is a watchword. The idea of RNA-based pest control strategies is highly popular since high species- and gene-specificity can be obtained at the level of the nucleic-acid sequence [131,255]. Currently, it is tempting to speculate that specificity of RNA-based insecticides could be achieved at additional levels.

First, several factors have been shown to influence the loading of sRNAs into Ago proteins, such as their sequence and structure [256,257,258,259,260,261,262,263,264]. In insects, generation of siRNAs with species-dependent length have been observed [265], and certain sRNA chemical modifications seem to vary among species. Specifically, *D. melanogaster* siRNAs are 2′-O-methylated, while this is not the case in the lepidopteran species *P. xylostella*, *B. mori,* and *Trichoplusia ni* [266,267]. In addition, RNAi genes have been shown to be fast evolving, resulting in lower levels of similarity between species (e.g., *dicer2* or *argonaute2*) [268,269,270]. It is therefore interesting to conjecture that a second level of specificity could be achieved, determined by a species-specific ability to intracellularly recognize and process the exogenous RNA molecules. Although research efforts are needed to explore these possibilities, one can hypothesize that this could be based on the length of sRNA molecules, its chemical modifications, and/or its complexation with specific (modified) proteins of the RNAi machinery.

Second, exhaustively unravelling mechanisms of RNA transfer within and between a wide range of organisms can stimulate the design of highly specific RNA delivery vehicles. It is important to elucidate what EV populations and protein-complexes interact with RNAs in different species, as well as to determine their composition and structure. It is also relevant to determine their specificity and, in this regard, an example has recently been reported in bees. Specifically, the domain of MRJP-3 that provides its RNA binding activity seems to have emerged in the *Apis* genus and to only be associated with jelly-secreting bees [72]. Moreover, efforts should be made to understand uptake mechanisms of these structures, including the potential involvement of specific ligand–receptor interactions. In this scope, specialized host–pathogen/symbiont interactions might be particularly useful, as they would further contribute to refine specificity and efficiency of RNA delivery. Noteworthily, RNA delivery strategies based on host–pathogen/symbiont systems have already been proposed, emphasizing the potential of these interactions [271,272,273,274]. It is inspiring to consider that, based on specialized uptake of certain optimized RNA-containing vehicles, the target species could be solely affected. 

In summary, three levels of specificity and efficiency could be aimed at, as determined by (1) the cellular delivery vehicle, (2) the recognition by the cellular machinery, and (3) the nucleotide sequence. Importantly, exploring these three levels in distinct contexts of integrated pest management has potential to delay, or avoid, the development of resistance. In addition, it is also relevant that the use of highly efficient and specific RNA delivery methods can contribute to the release of low quantities of the RNA-based insecticide in the environment, reducing potential toxicity concerns.

## 5. Conclusions

RNA molecules and their related pathways are classically described at the intracellular level. Recently, the existence of a novel RNA-based communication layer, via the spread of extracellular RNAs in biological fluids, became well established and gave rise to a novel research area that is currently booming in biomedical sciences. Although this research field remains underexplored in insects, growing evidence indicates that this route of communication is also functional in these animals. Specifically, extracellular RNAs are present in insects, are contained within EVs and/or interact with RBPs, and are transferred to recipient cells to exert their function. Nevertheless, only a limited number of studies are currently available, which do not cover the dimension and diversity of the class Insecta, or of the wide range of insect–plant interactions. A wider and deeper fundamental understanding of the intercellular RNA transfer mechanisms in these organisms is required. In this scope, there is an urgent need for well described, robust, and reproducible protocols for isolation of extracellular components, such as EVs and RBPs, from complex biofluids. In addition, although the advent of deep sequencing technologies has led to an increase of descriptive studies, these only form a starting point. In fact, functional studies regarding natural RNA-transfer mechanisms, as well as their potential for bioactive delivery of exogenous RNA molecules, are required. Noteworthily, it is critical to not only unravel conserved processes, but also species-specific mechanisms. Thus, besides model organisms, efforts must also be invested into several pest and beneficial systems. At last, on the long run, special attention should be given to the development of production/purification techniques of selected extracellular components bound to RNAs of interest; as well as to biosafety assays, namely concerning the specificity of the developed approaches.

Considering the potential of RNA-based insect pest control strategies, understanding the mechanisms by which RNAs are transferred within insects, as well as between plants and insects, promises to give leads to the development of effective strategies for exogenous RNA delivery. This has potential to lead to novel ways to protect crops, to combat insect pests, and to generate high economic, environmental and social value.

## Figures and Tables

**Table 1:** Summary of the reported studies demonstrating extracellular RNAs and their functional transfer in insects. Plasma refers to cell-free hemolymph. * Indicates that functionality was reported.

	Insect	Sample	RNA	Reference
Intercellular / Intraindividual	Korean rhinoceros beetle, *Allomyrina dichotoma*	plasma	sRNAs	[67]
	Fruit fly, *D. melanogaster*	plasma	miRNAs	[62]
	Fruit fly, *D. melanogaster*	medium of cultured cells (S2R+ and D17-c3)	sRNAs	[63]
	Fruit fly, *D. melanogaster*	medium of cultured cells (S2 and Cl8 cell lines)	miRNAs	[65]
	Fruit fly, *D. melanogaster*	plasma	siRNAs *	[64]
	Red flour beetle, *T. castaneum*	medium of cultured cells (TcA cell line)	siRNAs *, miRNAs and other sRNAs	[66]
	Colorado potato beetle, *L. decemlineata*	medium of cultured cells (Lepd-SL1 cell line)	dsRNA	[68]
Interindividual	Honey bee, *A. mellifera*	nurse bee secretions	miRNAs *	[70]
	Honey bee, *A. mellifera*	hemolymph, worker and royal jellies	dsRNA *, ssRNA and sRNAs	[71,72]
	Florida carpenter ant, *C. floridanus*	trophallactic fluid	miRNAs	[73]
Interspecies	Mosquito, *Aedes aegypti* and *Aedes albopictus*	saliva	miRNAs *	[60]
	Mosquito, *Anopheles coluzzii*	saliva	miRNAs and other sRNAs	[61]
	Parasitic wasp, *C. vestalis*	host body (Diamondback moth, *P. xylostella*)	miRNAs *	[74]

**Table 2:** Summary of the reported studies indicating natural RNA transfer from plant to insects. * Indicates that functionality was reported.

Plant	Insect	Sample	RNA	Reference
*C. melo*	Cotton-melon aphid, *A. gossypii*	whole insect	miRNAs	[107]
*Morus notabilis*	Silkworm, *B. mori*	hemolymph, fat body, and silk gland	miRNAs	[108]
*Sorghum bicolor*	Greenbug, *S. graminum*	whole insect	miRNAs	[109]
*Hordeum vulgare*	Yellow sugarcane aphid, *S. flava*	whole insect	miRNAs	[109]
*B. oleracea*	Green peach aphid, *M. persicae*	gut	miRNAs	[110]
*Arabidopsis thaliana*	Diamondback moth, *P. xylostella*	hemolymph	miRNAs *	[111]
*Brassica campestris*	Honey bee, *A. mellifera*	beebread and royal jelly	miRNAs *	[113]

## Data Availability

This article reviews published studies.
